# Burden of group A rotavirus infection among children with acute diarrhoea in Lambaréné, Gabon

**DOI:** 10.4102/jphia.v17i1.1617

**Published:** 2026-02-04

**Authors:** Bayode Romeo Adegbite, Jeannot Fréjus Zinsou, Jean Claude Dejon–Agobé, Jean Ronald Edoa, Yabo Josiane Honkpéhèdji, Michele Marion Ntsame Owono, Fabrice Beral M’Baidiguim, Gédéon Prince Manouana, Andréa Rosine Oméra Obele Ndong, Ayôla Akim Adegnika

**Affiliations:** 1 Centre de Recherches Médicales de Lambaréné (CERMEL), Lambaréné, Gabon; 2 Institut für Tropenmedizin, Eberhad Karls Universität Tübingen, Tübingen, Germany; 3 Department of Infectious Diseases, Division of Internal Medicine, Center of Tropical Medicine and Travel Medicine, Amsterdam University Medical Center, University of Amsterdam, Amsterdam, the Netherlands; 4 Leiden University Center for Infectious Diseases (LUCID), Leiden University Medical Center (LUMC), Leiden, the Netherlands; 5 Department of Infectiology, University Hospital, Libreville, Gabon; 6 German Center for Infection Research (DZIF), Partner Site Tübingen, Tübingen, Germany

**Keywords:** rotavirus infection, acute diarrhoea, children under five, prevalence, risk factors, Gabon, vaccination policy

## Abstract

**Background:**

Rotaviruses are among the most common causal pathogens of severe dehydrating diarrhoea in children. Little is known about the burden of rotavirus diarrhoea in Gabon.

**Aim:**

This study aimed to determine the proportion of rotavirus infection in children under 5 years with diarrhoea in Lambaréné and seen at the hospital and factors associated with rotavirus infection.

**Setting:**

The data used in this study were collected between February 2020 and February 2021 in children presenting with acute diarrhoea in the Albert Schweitzer Hospital paediatric ward.

**Methods:**

A cross-sectional study was carried out. Stool samples were tested for rotavirus antigens using the rotavirus Standard Diagnostic (SD) BIOLINE Rota and Adeno enzyme immunoassay detection kit.

**Results:**

A total of 178 children were included in the study. The proportion of rotavirus infection was 22% (*n* = 39/178; 95% confidence interval [CI]: 16% – 29%). In the multivariate analysis, the rotavirus was independently associated with dehydration (adjusted odds ratio [aOR] = 2.65; 95% CI: 1.09–6.86), vomiting (aOR = 3.15; 95% CI: 1.29–8.25), lethargy (aOR = 3.12; 95% CI: 1.16–8.71) and hospitalisation (aOR = 4.63; 95% CI: 1.7–13.65).

**Conclusion:**

Rotavirus infection was associated with severe diarrhoea and hospitalisation. This study shows the need to integrate and support free rotavirus vaccination into the expanded vaccination programme in Gabon.

**Contribution:**

This study provides evidence that could guide public health strategies and inform vaccine policies that could ultimately reduce the burden of rotavirus-associated diarrhoea in children.

## Introduction

Globally, rotaviruses are among the leading causes of severe diarrhoea in children under 5 years, accounting for approximately 25 million outpatient visits and more than 2 million hospitalisations each year, with the vast majority occurring in developing countries.^1^ In sub-Saharan Africa (SSA), the proportion of rotavirus diarrhoea in a hospital setting among children under 5 years is reported to vary between 11% and 45%.^2,3,4,5,6^ These figures highlight the uneven burden of disease and the urgent need for targeting interventions in high-risk regions. The World Health Organization (WHO) has recognised the critical role of vaccination in reducing the morbidity and mortality associated with rotavirus infection. As a result, WHO strongly recommends the inclusion of rotavirus vaccination in all expanded programmes of immunisation (EPI), prioritising countries in South and Southeast Asia and SSA. Four live attenuated oral rotavirus vaccines: Rotarix, RotaTeq, Rotavac and RotaSiil are available internationally and recommended by WHO for their efficacy and safety in preventing severe gastrointestinal diseases caused by rotavirus.^7,8^ Many countries, including Gabon, have yet to integrate rotavirus vaccines into their national EPI. In Gabon, diarrhoea remains a leading cause of medical consultations among children under 5 years old.^9^

While rotavirus is a well-recognised pathogen responsible for severe diarrhoea worldwide, its specific contribution to the burden of diarrhoea in Gabon, particularly in Lambaréné, remains inadequately documented. This knowledge gap presents a significant obstacle to the design and implementation of effective prevention and control measures. Furthermore, the limited published epidemiological data on rotavirus in Lambaréné limit our understanding of its role in diarrhoeal disease and its associated complications. A previous study in 2019 reported that rotaviruses are among the top five pathogens causing diarrhoea in young children presenting to the hospital in Lambaréné but lacked characterisation of clinical complications and socio-demographic associated factors that could help inform improved management and prevention.^10^

Given this context, there is a need for studies that document the proportion and associated factors of rotavirus infection in children. Such research is particularly critical in areas such as Lambaréné, where the epidemiology of rotavirus and its clinical implications are not well understood. Understanding these factors could guide public health strategies, inform vaccine policies and ultimately reduce the burden of rotavirus-associated diarrhoea in vulnerable populations. This study aims to determine the proportion of rotavirus infection and identify associated factors among children under 5 years old in a hospital setting in Lambaréné.

## Research methods and design

### Study design and setting

This was a cross-sectional study. The data were collected from 01 February 2020 to 28 February 2021, focused on the assessment of rotavirus infection in children presenting with acute diarrhoea at the Albert Schweitzer Hospital (HAS). All children with diarrhoea who come to the hospital are seen in the paediatric ward. A full-year recruitment period was chosen to cover all seasons (dry and rainy) in the region. Lambaréné is a small town in Gabon,^11^ it is located 240 km (by road) southeast of Libreville (the capital of Gabon). Lambaréné’s climate is equatorial: hot and humid. The year begins with a dry season between January and February, followed by a rainy season between March and May, a dry season from June to September and finally a rainy season from October to December.

### Inclusion and exclusion criteria

All children under 5 years with acute diarrhoea (characterised by loose, watery stools that typically last for a short duration, up to 2 weeks^12^ consulting in the paediatric ward were eligible. Neonates and children whose parents refused to provide informed consent to participate were excluded.

### Data collection and study procedure

After explaining the study to parents and/or legal guardians, informed consent was signed, and all children meeting the inclusion criteria were included in the study. The medical investigator collected socio-demographic information (age, sex, number of persons living in the same room, education level of mother or guardian) and vaccination status using a data collection form. Clinical examination was performed to evaluate hydration and neurological status and the presence of fever (which was defined as an axillary temperature exceeding 37.5 °C on admission). Stool specimens were collected on the day of enrolment using a sterile container and sent to the microbiology laboratory for analysis on the same day. Stool samples were processed using an immunochromatographic assay detection kit, SD BIOLINE Rota and Adeno (Standard Diagnostic, Inc.), Suwon city, Korea, according to the manufacturer’s instructions. The Standard Diagnostic (SD) BIOLINE Rota and Adeno test has a sensitivity of 94% and specificity of 98.3%.^13^

### Sample size

The estimated sample size of the study was 152 children under 5 years, calculated based on the minimum proportion of rotavirus expected of 11%^3,14^ among children under 5 years with acute diarrhoea in the region: confidence interval (CI) of 95% (type value 1.96) and random error of 5% (type value of 0.05). The sample size was determined using EPI Info software.^15^

### Data management and statistical analysis

The data entered in the ‘Redcap’ database was exported with R Studio software version 3.6.1 (Foundation for Statistical Computing, Vienna, Austria) for clearance and statistical analysis. The normality of the quantitative variables was assessed using the Shapiro–Wilk test and visual inspection of the histogram. To identify the factors associated with rotavirus infection, we categorised them into two groups: socio-demographic and clinical factors. Firstly, a bivariate analysis of the variable ‘rotavirus’ with demographic and clinical characteristics was made. Secondly, we performed the multivariate analysis (logistic regression). The socio-demographic and clinical variables with a *p*-value less than 0.2 during the bivariate analysis were included in the multivariable model. We added to this the variables (children’s age, gender, source of drinking water and fever), which did not meet the 20% *p*-value rate at the bivariate level but were described in the literature as associated with rotavirus infection. Crude odds ratio (COR) and adjusted odds ratios (aOR) were generated, as well as their 95% CI. We checked the adequacy of the model using the Hosmer and Lemeshow test (goodness-of-fit test). The model adequacy test concluded that there was a good adequacy with a *p*-value = 0.888.

### Ethical considerations

Ethical clearance to conduct this study was obtained from the Lambaréné Medical Research Center Institutional Research Ethics Committee (No. 003/2017). Parents or guardians were requested to sign a consent form for their willingness to participate in the study.

## Results

### Socio-demographic characteristics of study participants

A total of 201 parents or guardians’ children were approached for the study, and 178 agreed to have their children included. The median age of the children was 12 months, with an interquartile range (IQR) of 7–18 months. None of the children received the rotavirus vaccine. The mothers or guardians of the children who had diarrhoea during the study period were aged 18–48 years. The majority (56%, *n* = 100/178) of these mothers were young adults between 18 years and 25 years of age. The peak of rotavirus proportion was observed during the dry season.

### Proportion, socio-demographic and clinical factors associated with rotavirus infections

The proportion of rotavirus infection in children who provided stool samples was 22% (*n* = 39/178; 95% CI: 16% – 29%). In a bivariate analysis, children from mothers between the ages of 26 years and 30 years, compared to those between the ages of 31 years and 48 years, were more likely to have rotavirus in their stools (OR = 2.38; 95% CI: 1.87–7.13), as shown in Table 1. Children whose mother had at least secondary education were less likely to have rotaviruses in their stool (OR = 2.51, 95% CI: 1.16–5.83). The associated clinical factors were: lethargy (OR = 2.85; 95% CI: 1.34–6.04), dehydration (OR = 2.32; 95% CI: 1.11–5.14) and hospitalisation (OR = 2.54; 95% CI: 1.23–5.35) (Table 2).

**TABLE 1:** Socio-demographic factors associated with rotavirus infection in children under 5 years in Lambaréné (bivariable analysis compared to children with diarrhoea but not rotavirus).

Variables	Rotavirus negative (*n* = 139)		Rotavirus positive (*n* = 39)		Odds ratio	95% CI	Overall *p*-value
	*n*	%	*n*	%			
**Child age group (months)**	-	-	-	-	-	-	0.590
13–59	67	48.2	21	53.8	Reference	-	-
7–12	40	28.8	8	20.5	0.65	0.25; 1.56	-
1–6	32	23.0	10	25.6	1.00	0.41; 2.35	-
**Maternal or guardian age group (years)**	-	-	-	-	-	-	0.040
31–48	27	19.4	7	17.9	Reference	-	-
26–30	27	19.4	17	43.6	2.38	1.87; 7.13	-
18–25	85	61.2	15	38.5	0.68	0.25; 1.96	-
**Sex of children**	-	-	-	-	-	-	0.638
Female	58	41.7	14	35.9	Reference	-	-
Male	81	58.3	25	64.1	1.27	0.61; 2.73	-
**Maternal or guardian level of education**	-	-	-	-	-	-	0.029
Secondary and more	65	46.8	10	25.6	Reference	-	-
Primary	74	53.2	29	74.4	2.51	1.16; 5.83	-
**Source of drinking water**	-	-	-	-	-	-	0.358
River well	45	32.4	9	23.1	Reference	-	-
Tap water (public or private)	94	67.6	30	76.9	1.58	0.71; 3.81	-
**Room crowding**	-	-	-	-	-	-	-
2–4 per room	76	54.7	21	53.8	Reference	-	-
> 4 per room	63	45.3	18	46.2	1.03	0.50; 2.12	-
**Type of toilet**	-	-	-	-	-	-	0.067
Other	34	24.5	16	41.0	Reference	-	-
Water-closet	105	75.5	23	59.0	0.47	0.22; 1.00	-
**Residence area**	-	-	-	-	-	-	0.387
Rural	46	33.1	10	25.6	Reference	-	-
Urban	93	66.9	29	74.4	1.42	0.65; 3.32	-
**Season**	-	-	-	-	-	-	0.033
Rainy	58	41.7	9	23.1	Reference	-	-
Dry	81	58.3	30	76.9	2.35	1.07; 5.65	-

CI, confidence interval.

**TABLE 2:** Clinical factors associated with rotavirus infection in children under 5 years in Lambaréné (bivariable analysis compared to children with diarrhoea but not rotavirus).

Variables	Rotavirus negative (*n* = 139)		Rotavirus positive (*n* = 39)		Odds ratio	95% CI	Overall *p*-value
	*n*	%	*n*	%			
**Fever**	-	-	-	-	-	-	0.892
No	61	43.9	16	41.0	Reference	-	-
Yes	78	56.1	23	59.0	1.12	0.55; 2.35	-
**Hospitalisation**	-	-	-	-	-	-	0.017
No	89	64.0	16	41.0	Reference	-	-
Yes	50	36.0	23	59.0	2.54	1.23; 5.35	-
**Vomiting**	-	-	-	-	-	-	0.091
No	77	55.4	15	38.5	Reference	-	-
Yes	62	44.6	24	61.5	1.97	0.96, 4.17	-
**Dehydration**	-	-	-	-	-	-	0.039
Absent	71	51.1	12	30.8	Reference	-	-
Present	68	48.9	27	69.2	2.32	1.11; 5.14	-
**Lethargy**	-	-	-	-	-	-	0.008
No	107	77.0	21	53.8	Reference	-	-
Yes	32	23.0	18	46.2	2.85	1.34; 6.04	-

CI, confidence interval.

### Multivariate analysis

Independent clinical presentations associated with rotavirus infection were using water-closet toilets (aOR = 0.38; 95% CI: 0.15–0.94), dehydration aOR = 2.65; 95% CI: 1.09–6.86), vomiting (aOR = 3.15; 95% CI: 1.29–8.25), lethargy (aOR = 3.12; 95% CI: 1.16–8.71) and hospitalisation (aOR = 4.63 (95% CI: 1.7–13.65), as shown in Table 3.

**TABLE 3:** Socio-demographic and clinical factors associated with rotavirus infection in children under 5 years (multivariate analysis).

Variables	Adjusted odds ratio	95% CI	*p*-value
**Child age group (months)**			
13–59	Reference	-	-
7–12	0.60	0.19–1.82	0.381
0–6	2.07	0.69–6.21	0.191
**Mother’s age group (year)**			
31–48	Reference	-	-
26–30	2.36	0.70–8.77	0.177
18–25	0.87	0.26–3.21	0.822
**Mother’s level of education**			
Secondary and more	Reference	-	-
Primary	2.41	0.88–7.18	0.097
**Sex**			
Female	Reference	-	-
Male	1.34	0.55–3.42	0.523
**Season**			
Rainy	Reference	-	-
Dried	0.92	0.33–2.63	0.866
**Type of Toilet commonly used**			
Other	Reference	-	-
Water-closet	0.38	0.15–0.94	0.037
**Hospitalisation**			
No	Reference	-	-
Yes	4.63	1.70–13.72	0.004
**Dehydration**			
Absent	Reference	-	-
Present	2.65	1.09–6.86	0.036
**Fever**			
No	Reference	-	-
Yes	1.40	0.48–4.23	0.539
**Vomiting**			
No	Reference	-	-
Yes	3.15	1.29–8.25	0.014
**Lethargy**			
No	Reference	-	-
Yes	3.12	1.16–8.71	0.026

CI, confidence interval.

## Discussion

Our study reports for the first time the hospital-based proportion of cases with acute diarrhoea caused by rotavirus and associated factors with rotavirus among children under 5 years with diarrhoea in Lambaréné. The proportion of rotavirus infection in children under 5 years with diarrhoea in Lambaréné was found to be 22% (95% CI: 16% – 29%). The independent factors associated were: using water-closet toilets, vomiting, lethargy, dehydration and hospitalisation.

The hospital proportion of rotavirus infection in Lambaréné is comparable to that carried out in neighbouring Cameroon and Tanzania in East Africa in 2017, where they reported a proportion of 21% and 26%, respectively, in children under 59 months^16,17^ in hospital setting However, the proportion in our study is lower than that reported in Uganda (45%)^18^ and Kenya (37%).^3^ The difference could be explained by the period of data collection. Indeed, the studies in Kenya and Uganda were conducted during the peak period of rotavirus infection.

Children whose mothers or guardians used water-closet toilets had a lower OR of having a rotavirus infection, suggesting that improved sanitation may play a protective role. However, demonstrating the direct impact of water-closet toilets and consistent hand hygiene practices by parents and guardians remains challenging, as their behaviours are often self-reported and subject to variability. Strengthening hygiene education and access to improved sanitation facilities could therefore be an important complementary strategy in reducing rotavirus transmission.

Our results indicate that the clinical signs associated with rotavirus are vomiting, lethargy and dehydration. The combination of these signs explains the severity of rotavirus infection. Furthermore, rotavirus infection was significantly associated with hospitalisation compared to acute diarrhoea cases not caused by rotavirus, highlighting its impact on the health service.

Notably, no child in our study had been vaccinated against rotavirus. This finding underscores the need for free rotavirus vaccine introduction into the Gabon EPI. Evidence from multiple studies demonstrates that countries that have integrated rotavirus vaccination for free into their EPI schedules have achieved substantial reduction in morbidity and mortality of diarrhoea among children under 5 years.^19,20^

### Strengths and limitations

Our study best describes the hospital-based proportion of rotavirus diarrhoea in Lambaréné. Our study is of public health interest; it determines the proportion of rotavirus diarrhoea before the possible introduction of the rotavirus vaccine in the EPI in Gabon. This study is the first, to our knowledge, to describe the factors associated with rotavirus in children under five in Lambaréné. However, some limitations are to be noted. The cross-sectional design of our study did not allow the collection of additional follow-up data. It would have been interesting to follow up with children to assess the outcome of treatment and mortality related to rotaviruses in Lambaréné. In addition, the causal relationship between diarrhoea and the presence of rotaviruses cannot be established in our study, and we cannot exclusively associate clinical signs with rotaviruses alone because the other pathogens responsible for gastroenteritis in children were not assessed. No genotyping analysis was performed to determine the serotype and genotype of rotavirus. This was also a hospital-based study, so we may have missed a lot of non-hospitalised cases. Despite these limitations, our study greatly contributes to enriching the epidemiology of rotavirus infection in SSA, especially in central Africa, where data on the proportion of rotavirus infection are scarce. Our study also opens up research perspectives on the morbidity and cost-effectiveness of the introduction of the rotavirus vaccine could have among children in Gabon

## Conclusion

Our study demonstrates the proportion of rotavirus diarrhoea among children under 5 years in a hospital in Lambaréné. The rotavirus infection is associated with the hospitalisation of children with acute diarrhoea. None of the children received the rotavirus vaccine. This calls for the free introduction of the rotavirus vaccine into the expanded vaccination programme in Gabon to reduce the morbidity of rotavirus diarrhoea in Gabon.
